# Supplementary material for: Quality of life of older Chinese adults receiving primary care in Wuhan, China: a multi-center study
Source: PeerJ. 2019 Apr 30;7:e6860. doi: 10.7717/peerj.6860 (PMC6499053; doi:10.7717/peerj.6860)
Supplement: Table S1 — The scale has six questions and each assesses one domain of quality of life (physical health, psychological health, economic circumstances, activities, family relationship, and relationships with non-family associates) on a five-point scale: 1=very poor, 2=poor, 3=fair, 4=good, 5=very good. [file peerj-07-6860-s001.docx]

**The Chinese six-item Quality of Life Scale**

**The following questions ask how you feel about your quality of life in the last four weeks. Please choose the answer that appears most appropriate. Please keep in mind that we ask that you think about your life in the last four weeks.**

| 1. How would you rate your physical health? | Very poor | Poor | Fair | Good | Very good |
| --- | --- | --- | --- | --- | --- |
| 2. How would you rate your mental health? | Very poor | Poor | Fair | Good | Very good |
| 3. How would you rate your economic conditions? | Very poor | Poor | Fair | Good | Very good |
| 4. How would you rate your activities? | Very poor | Poor | Fair | Good | Very good |
| 5. How would you rate your relationship with family members? | Very poor | Poor | Fair | Good | Very good |
| 6. How would you rate your relationship with others? | Very poor | Poor | Fair | Good | Very good |
